# Supplementary material for: Endoscopic ultrasound-guided portosystemic pressure gradient measurement vs. transjugular balloon occlusion measurement in patients with cirrhosis (ENCOUNTER): A bicentric EU study
Source: JHEP Rep. 2025 May 29;7(8):101466. doi: 10.1016/j.jhepr.2025.101466 (PMC12276445; doi:10.1016/j.jhepr.2025.101466)
Supplement: Multimedia component 1 [file mmc1.pdf]

Endoscopic ultrasound-guided portosystemic pressure gradient measurement *vs.* transjugular balloon occlusion measurement in patients with cirrhosis (ENCOUNTER): A bicentric EU study

Emma Vanderschueren, Wim Laleman, Lawrence Bonne, Geert Maleux, David R. Wagner, Chyon Yeh, Andrea Calvo, Oriol Sendino, Angels Gines, Anna Baiges, Marco J. Bruno, Juan Carlos Garcia-Pagan, Schalk van der Merwe

Table of contents

Table S1……………………………………………………………………………..2

Table S2……………………………………………………………………………..3

Table S3……………………………………………………………………………..4

Table S4……………………………………………………………………………..4

Fig. S1……………………………………………………………………………….5

Fig. S2……………………………………………………………………………….5

Fig. S3……………………………………………………………………………….6

Table S1. Study exclusion criteria

**General criteria**	Patient is < 18 or > 85 years of age
	Patient is pregnant, breast-feeding, or planning to become pregnant during the course of the study
	Patient is unwilling or unable to sign and date the informed consent
	Patient is unwilling to comply with the follow-up study schedule
	Patients for whom endoscopic procedures are contraindicated
	Patients for whom propofol general anesthesia is contraindicated
**Medical criteria**	Platelet count <50,000 per mm3
	INR > 1.7
	eGFR < 50 mL/min/1.73m2
	Previous transjugular intrahepatic or surgical portosystemic shunt
	Previous total or partial splenectomy
	Non-cirrhotic portal hypertension
	SBP within the last three months irrespective of treatment for SBP
	Patients with known infection which is not controlled by medical intervention
	Portopulmonary hypertension
	Cardiac decompensation
	Pre-sinusoidal liver disease
	Cholestatic liver disease
	Patient who received endoscopic treatment for upper gastrointestinal variceal bleeding within the past 7 days
	Patients with current HCC
**Anatomical criteria***	Portal vein thrombosis
	Anatomic abnormalities of the hepatic vasculature that prevent access to the intrahepatic portion of the portal vein or hepatic veins
	Evidence of active gastrointestinal bleeding
	If the volume of ascites in the path of the needle prevents apposition of the gastrointestinal tract and liver

* Identified at screening and/or during the endoscopic procedure
Abbreviations: INR, international normalized ratio; eGFR, estimated glomerular filtration rate; SBP, spontaneous bacterial peritonitis; HCC, hepatocellular carcinoma

Table S2. Primary and secondary study objectives

**Primary study objective**
Correlation between EUS-PPG obtained using the EchoTip® Insight™ and the HVPG performed simultaneously under general anesthesia
**Secondary study objective**
a. Comparison of the EUS-guided PVP (using the EchoTip® Insight™) to the transjugular WHVP
b. Comparison of the EUS-guided HVP (using the EchoTip® Insight™) to the transjugular FHVP
c. Evaluation of technical success (ability to take three measurements of the HVP and PVP using the EchoTip® Insight™ system)
d. Procedure-related adverse events
e. Evaluation of the association between EUS-guided measurements with clinical and lab-based features of portal hypertension such as varices, portal hypertensive gastropathy, thrombocytopenia, and liver elastography (if available within 1 year prior to procedure).
f. <u>For patients undergoing TIPS placement:</u> EUS-guided PVP (using the EchoTip® Insight™) is compared to the transjugular direct PVP obtained during the procedure
Abbreviations: EUS, endoscopic ultrasound; PPG, portal pressure gradient; HVPG, hepatic venous pressure gradient; PVP, portal venous pressure; WHVP, wedged hepatic venous pressure; HVP, hepatic venous pressure; FHVP, free hepatic venous pressure; TIPS, transjugular intrahepatic portosystemic shunt

Table S3. Differences in pressures depending on location of EUS-guided pressures.

	**EUS-PVP measured in the**		
	**Left portal vein branch (n = 13)**	**Main portal vein (n = 8)**	p-value*
EUS-PVP	24.6 ± 6.5	22.4 ± 5.3	0.42
EUS-PPG	11.2 ± 6.6	10.3 ± 3.5	0.71
	**EUS-HVP measured in the**		
	**Left hepatic vein (n = 11)**	**Middle hepatic vein (n = 10)**	p-value*
EUS-HVP	13.2 ± 3.8	12.6 ± 4.4	0.73
EUS-PPG	11.6 ± 6.3	10.0 ± 4.7	0.51

*Comparison of variances was performed using the F-test.
Numbers represent means ± standard deviations.
Abbreviations: EUS, endoscopic ultrasound; PPG, portal pressure gradient; PVP, portal venous pressure; HVP, hepatic venous pressure

Table S4: Disparity in hepatic venous pressure measurements (post hoc analysis)

	**|HVP − FHVP| < 3 mmHg** n = 10	**|HVP − FHVP| ≥ 3 mmHg** n = 11	**p-value***
**Baseline variables**			
Child-Pugh score	6.6 ± 1.7	8.2 ± 1.9	0.06
MELD score	9.5 ± 2.0	11.9 ± 5.4	0.43
BMI	27.4 ± 7.4	24.7 ± 4.7	0.34
**Hepatic vein GI access point (EUS)**			
Left hepatic vein	70.0%	36.4%	0.12
Middle hepatic vein	30.0%	63.6%	
**Hepatic vein assessment (EUS)**			
Depth of liver parenchyma traversed (mm)	36.6 ± 18.5	30.9 ± 8.8	0.38
Distance between hepatic vein puncture and IVC ostium	22.4 ± 8.1	14.2 ± 10.5	0.09
Diameter of the target segment of the hepatic vein (mm)	4.8 ± 1.8	7.1 ± 9.1	0.44

* Unadjusted raw p-value. All p-values in the current table are not statically significant after correction using the Hochberg procedure.
Numbers represent means ± standard deviations.
Abbreviations: HVP, hepatic venous pressure; FHVP, free hepatic venous pressure; MELD, model of end stage liver disease; BMI, body mass index; TIPS, transjugular portosystemic shunt; GI, gastrointestinal; EUS, endoscopic ultrasound; IVC, inferior vena cava

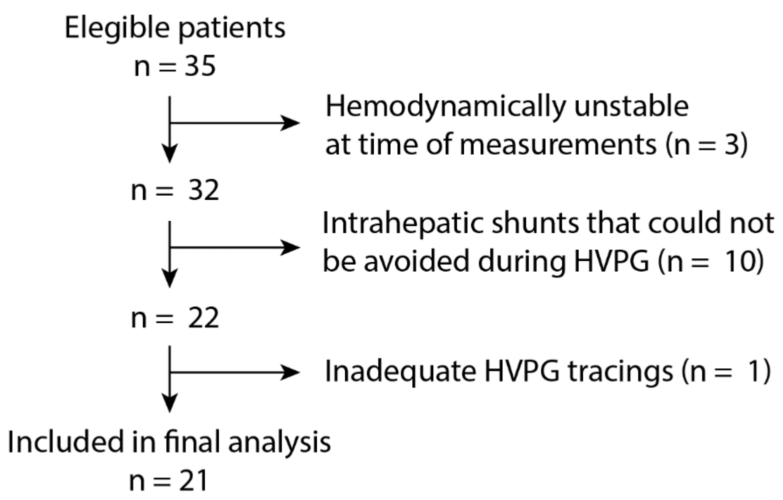

Fig. S1. Inclusion and exclusion flowchart

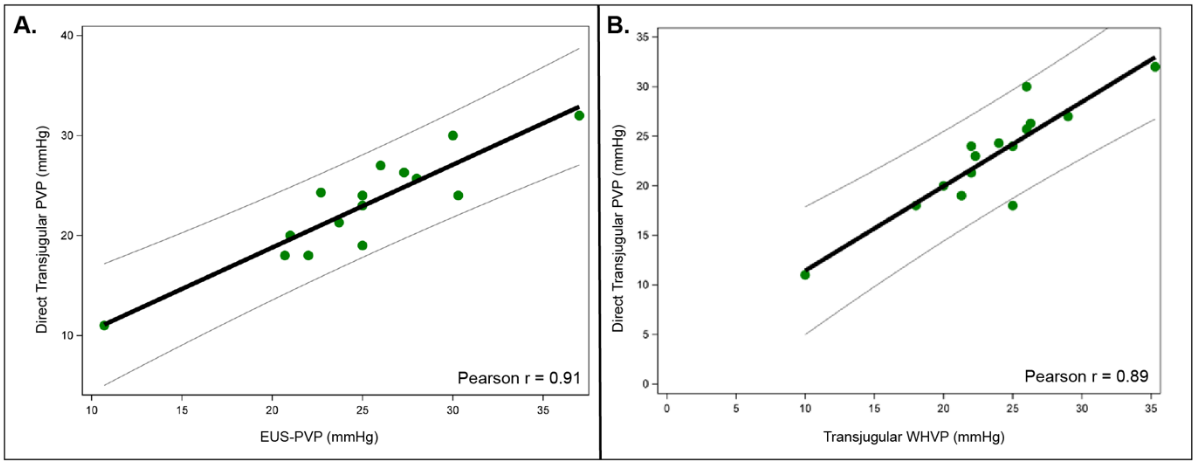

Fig. S2. (A) Correlation of direct transjugular portal pressure vs. EUS-PVP in patients receiving TIPS, Pearson r = 0.91, p <0.0001; (B) Correlation of the direct transjugular portal pressure vs. transjugular WHVP in patients receiving TIPS, Pearson r = 0.89, p <0.0001.

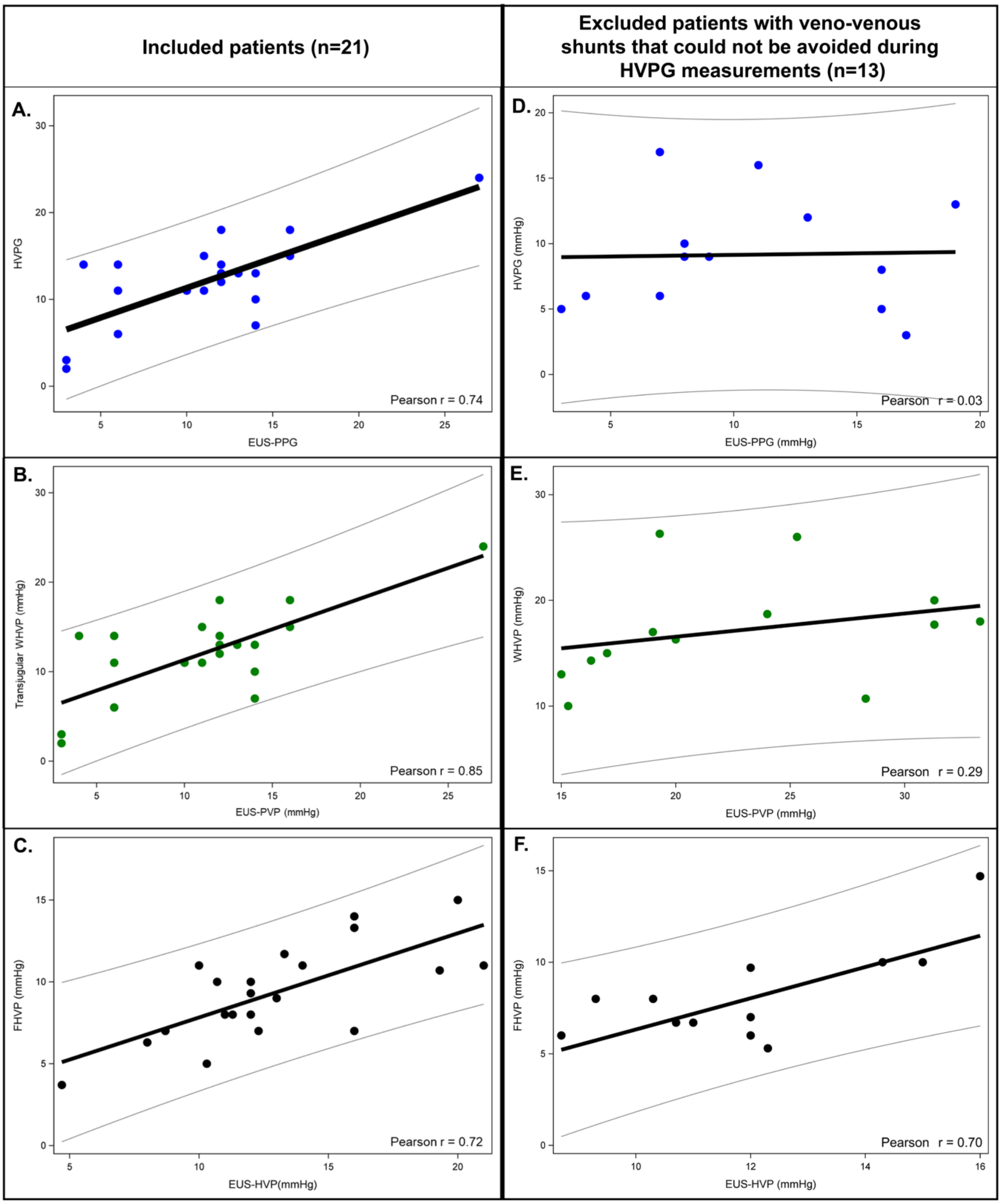

Fig. S3. Comparison of Pearson correlations for included patients (A, B and C) and patients excluded because of intrahepatic veno-venous shunts that could not be avoided during HVPG measurements (D, E, F) (all pressure were measured under general anesthesia). (A/D) HVPG vs EUS-PPG, (B/E) transjugular WHVP vs. EUS-PVP, (C/F) transjugular FHVP vs EUS-HVP.
