# Supplementary material for: Endoscopic ultrasound-guided portosystemic pressure gradient measurement vs. transjugular balloon occlusion measurement in patients with cirrhosis (ENCOUNTER): A bicentric EU study
Source: JHEP Rep. 2025 May 29;7(8):101466. doi: 10.1016/j.jhepr.2025.101466 (PMC12276445; doi:10.1016/j.jhepr.2025.101466)
Supplement: Multimedia component 4 [file mmc4.pdf]

Endoscopic ultrasound-guided portosystemic pressure gradient measurement *vs.* transjugular balloon occlusion measurement in patients with cirrhosis (ENCOUNTER): A bicentric EU study

Emma Vanderschueren[1,2,*], Wim Laleman[1,3,‡], Lawrence Bonne[4], Geert Maleux[4], David R. Wagner[5], Chyon Yeh[6], Andrea Calvo[7], Oriol Sendino[8], Angels Gines[9], Anna Baiges[10], Marco J. Bruno[11], Juan Carlos Garcia-Pagan[10,‡], Schalk van der Merwe[1,2,*,‡]

JHEP Reports **2025**. vol. 7 | 1–10

Check for updates

Background & Aims: Patients with cirrhosis and portal hypertension are at increased risk of hepatic decompensation and liver-related mortality. While the hepatic venous pressure gradient (HVPG) is the accepted method for quantifying portal hypertension, its measurement and limited availability pose challenges. Endoscopic ultrasound-guided portal pressure gradient (EUS-PPG) provides a direct alternative. The ENCOUNTER study is the first to compare EUS-PPG to HVPG in the same patient, simultaneously.

Methods: This prospective, international, bicentric study included patients referred for HVPG or transjugular intrahepatic portosystemic shunt (TIPS) placement at the University Hospital of Leuven (Belgium) and Hospital Clinic Barcelona (Spain). Patients underwent standard-of-care HVPG, followed by simultaneous HVPG and EUS-PPG measurements under propofol general anesthesia.

Results: The final analysis included 21 patients with cirrhosis undergoing simultaneous HVPG and EUS-PPG measurements, of whom 15 received TIPS. Mean HVPG and EUS-PPG values under general anesthesia were comparable (11.9 ± 5.2 *vs.* 10.9 ± 5.6 mmHg, p = 0.2332) and showed good correlation (r = 0.74, p = 0.0001). The individual pressure components also showed a good correlation (portal vein: r = 0.85, p <0.0001; hepatic vein: r = 0.72, p = 0.0003). In patients receiving TIPS, direct transjugular portal pressure measurements demonstrated an excellent correlation with EUS-guided portal pressures (r = 0.91, p <0.0001). Technical success was achieved in all cases, with no adverse events associated with the EUS-PPG procedure.

Conclusion: EUS-PPG is a reliable and safe alternative to HVPG for the direct measurement of portal pressure. However, attention must be paid to technical challenges, including the potential overestimation of EUS-guided hepatic vein pressures and the impact of general anesthesia, which may alter pressure measurements and subsequently affect risk classification.

ClinicalTrials.gov: NCT04987034.

© 2025 The Authors. Published by Elsevier B.V. on behalf of European Association for the Study of the Liver (EASL). This is an open access article under the CC BY license (http://creativecommons.org/licenses/by/4.0/).

Introduction

Cirrhosis contributes to approximately two million deaths worldwide annually.[1] The progression to advanced cirrhosis is paralleled by a gradual increase in portal pressure, ultimately leading to the development of portal hypertension. This condition typically remains asymptomatic until it surpasses a critical threshold, known as clinically significant portal hypertension (CSPH), at which point complications such as variceal bleeding, ascites, hepatic encephalopathy and hepatocellular carcinoma may arise.[2] A timely diagnosis of CSPH is relevant because interventions that reduce portal pressure can prevent or delay these life-threatening complications. However, diagnosing CSPH can be challenging in patients with advanced but compensated chronic liver disease, where clinical signs of CSPH may not be apparent.

Traditionally, portal pressure is assessed via the hepatic venous pressure gradient (HVPG) which indirectly gauges portal pressure by wedging a balloon-tipped catheter in the right hepatic vein. An HVPG of ≥10 mmHg defines CSPH.[3–5] An abundance of evidence has underscored the prognostic value of HVPG and its role in guiding treatment for chronic liver disease.[2,6–8] Nevertheless, HVPG measurement has limitations, mostly stemming from the indirect nature of portal pressure assessment. It is unable to evaluate presinusoidal portal hypertension, which is characteristic of conditions such as non-cirrhotic portal hypertension, sarcoidosis, congenital hepatic

* Corresponding authors. Address: University Hospital Leuven campus Gasthuisberg, Department of Gastroenterology and Hepatology, Herestraat 49, 3000 Leuven, Belgium; Tel.: +32 16 34 42 99, fax: +32 16 34 43 87.
E-mail addresses: emma.vanderschueren@uzleuven.be (E. Vanderschueren), schalk.vandermerwe@uzleuven.be (S. van der Merwe).
‡ Co-senior authorship
https://doi.org/10.1016/j.jhepr.2025.101466

fibrosis, and to some extent, primary biliary cholangitis and metabolic dysfunction-associated steatotic liver disease (MASLD).[2,3] In patients with MASLD-related cirrhosis undergoing transjugular intrahepatic portosystemic shunt (TIPS) placement, discrepancies between the wedged hepatic venous pressure (WHVP) and direct portal venous pressure (PVP) measurements have been observed in up to 38% of cases.[9] HVPG will also underestimate portal pressure in the presence of intrahepatic veno-venous communications. These communications, which hinder the establishment of a static column upon balloon inflation, occur in 1% to 36% of patients. Their prevalence is notably higher in patients with advanced liver disease, affecting the accuracy of HVPG assessments.[10–13] Additionally, HVPG measurement is often only available in tertiary care centers and requires the use of ionizing radiation.

Recently, endoscopic ultrasound (EUS)-guided portal pressure gradient (EUS-PPG) measurement, which relies on direct venipuncture of the hepatic and portal veins via the gastrointestinal tract under EUS guidance, was developed.[14] EUS-PPG measurement was first described in an animal model in 2004, followed by the publication of the first human case report a decade later and quickly followed by the first human pilot study by Huang *et al.*[15–17] Since then, several studies have confirmed the feasibility and safety of the procedure and have demonstrated that EUS-PPG correlates well with histological hepatic fibrosis stage and indirect signs of CSPH.[18–23] The primary advantage of the EUS-guided approach to portal pressure measurement is the potential for a ‘one-stop procedure’. EUS-PPG can be combined with an evaluation of the liver parenchyma and adjacent tissues to detect signs of advanced liver disease (*i.e.* nodular liver surface, hypertrophic caudate lobe, ascites, liver nodules, portal vein thrombosis, splenomegaly, collaterals, varices, or portal hypertensive gastropathy). Additionally, it provides the option of a simultaneous EUS-guided liver biopsy, EUS-guided elastography or other (therapeutic) endoscopic procedures.[14]

However, despite these advantages, a direct comparison of EUS-guided and transjugular pressure measurements in patients with chronic liver disease has not been published. To address this gap, the ENCOUNTER study was designed as the first study to directly compare EUS-PPG with HVPG, performed in the same patient, simultaneously.

Patients and methods

Study design

This is an international, prospective, clinical post-marketing study. Patients were enrolled in the University Hospital of Leuven (Belgium) and Hospital Clinic Barcelona (Spain). All patients referred for a procedure involving measuring HVPG (such as transjugular liver biopsy or non-urgent TIPS) were considered for inclusion. Exclusion from enrollment was determined based on the criteria listed in Table S1. All patients read and signed the informed consent document. This study was conducted in accordance with the ethical principles of the Declaration of Helsinki, the ISO 14155 and the European Medical Device Directive (Council Directive 93/42/EEC) and was reviewed and approved by the Ethics Committees of both institutions. Patient enrollment started in July 2021 and continued until September 2023. All authors had access to the study data, and reviewed and approved the final manuscript.

Study procedure

Patient screening, including laboratory assessments, was performed within 28 days of the procedure. Liver elastography results were documented, if available. Measurements were performed using the FibroScan® device (Echosens, Paris, France) following the manufacturer’s recommendations. Both HVPG and EUS-PPG measurements were performed in the same patient simultaneously to ensure consistency in measurement conditions. To preserve the integrity of the study, the EUS endoscopist and interventional radiologist or hepatologist performing the HVPG measurements, as well as the respective teams, were blinded to the results of each other’s measurements during the procedure. All HVPG measurements were reviewed for quality by an independent physician not affiliated with the centers conducting the study (JA). Paracentesis was performed the day before or the morning of the study interventions to avoid fluid in the path of the EUS needle. Broad-spectrum prophylactic antibiotics were administered 1 h before the procedure. Patients were positioned supine, with the interventional radiologist positioned on the patient’s right side and the EUS endoscopist on the left. Transducers for both the EUS-guided and transjugular procedures were positioned at identical levels and aligned with the phlebostatic axis to ensure consistency in pressure measurements.

Simultaneous measurement of EUS-PPG and HVPG were performed under general anesthesia enhancing patient comfort and ensuring precise control. This setup allowed for the concurrent application of both techniques and maximized patient safety, in compliance with the ethical review board’s requirements. To account for any potential variations in portal pressure caused by anesthetic agents, an initial set of HVPG measurements was carried out with the patient awake, using only local anesthesia, in line with standard practice. Following these preliminary measurements, patients were given general anesthesia with propofol and fentanyl (derivatives), then intubated and placed on mechanical ventilation. Subsequently, they underwent simultaneous EUS-guided and transjugular pressure measurements. Throughout the procedure, continuous hemodynamic monitoring was overseen by an anesthesiologist.

For patients requiring TIPS, the same procedural sequence was followed, with both HVPG and EUS-PPG measurements taken prior to TIPS placement. Following these measurements, the right portal vein was accessed via puncture from a hepatic vein branch to establish the TIPS trajectory. Then the direct PVP readings were obtained in triplicate. The TIPS procedure was subsequently completed according to standard-of-care protocols. All patients were monitored for adverse events, with surveillance extending up to 30 days post-procedure.

HVPG measurements

HVPG was measured by an experienced physician (GM, LB, AB, JCGP) according to published guidelines.[3] Briefly, the right internal jugular vein is punctured under ultrasound guidance using local anesthesia. Then, a balloon-tipped catheter is advanced into the hepatic vein to measure the free hepatic venous pressure (FHVP). Next, the balloon is inflated and the WHVP is measured. Measurements were retrieved in triplicate and averaged.

EUS-PPG measurements

EUS procedures were performed by endoscopists with longstanding experience in diagnostic and therapeutic EUS (SV, WL, OS, AG). EUS-PPG measurements were obtained using the EchoTip® Insight™ device (Cook Ireland Ltd), which is an FDA-approved commercially available system that consists of a 25-gauge EUS needle, a small pressure transducer, and non-compressible connection tubing. The EchoTip® Insight™ system was assembled, calibrated, and used in accordance with the IFU (instructions for use).

After introduction of the EUS scope, a careful assessment for the presence of exclusion criteria was performed (Table S1 for anatomical exclusion criteria). Next, the pressure transducer was secured at the level of the phlebostatic axis, and the EchoTip® Insight™ was introduced through the scope. First, the hepatic vein was accessed using the needle via a transgastric approach, targeting the vein a few centimeters from its ostium into the inferior vena cava (IVC). Once introduced in the vessel the needle is flushed with saline, which is visible as bubbles on the EUS screen. The hepatic venous pressure (HVP) is recorded in triplicate, flushing the catheter with up to 0.5 ml in between each reading. Next, the intrahepatic portion of the portal vein is punctured via a transgastric (or transduodenal) approach. Similarly, the needle is flushed before each measurement and the PVP is recorded in triplicate. The needle is retracted from within the vessel into the liver parenchyma under Doppler ultrasound observation. Flow within the needle tract will indicate the presence of extravasation of blood. Once signs of extravasation are absent, the needle is retracted into the sheath and removed. EUS images were saved, and EUS-guided pressure measurements were video recorded.

Primary and secondary endpoints

The primary study objective was to evaluate the correlation between EUS-PPG measurements, obtained using the EchoTip® Insight™, and HVPG values, measured simultaneously under general anesthesia. Secondary aims included comparing the EUS-guided PVP to the transjugular WHVP, comparing the EUS-guided HVP to the transjugular FHVP, evaluating technical success and procedure-related adverse events, and assessing the association of EUS-guided measurements with clinical and laboratory-based features of portal hypertension. In patients undergoing TIPS placement, EUS-guided PVP was compared to the transjugular direct PVP obtained during the procedure. Technical success is defined as the ability to take three measurements of the HVP and PVP using the EchoTip® Insight™ system. Study objectives have been summarized in Table S2.

Sample size

The expected Pearson's correlation between the EUS-PPG and HVPG measurements under general anesthesia was 0.70 to 0.95. Based on the paper from Moinester and Gottfried,[24] a sample size of 12 would provide a 0.70 Pearson's correlation with a half-width range of 0.35. When the observed correlation value is larger than 0.70, we expected to see a narrower confidence interval with a sample size of 12 or more. If this was not the case after 30 enrolled patients, additional patients would be unlikely to result in a higher correlation. Therefore, the minimum sample size was set at 12, with a maximum of 30. To account for the inability to obtain access to the intrahepatic portion of either the portal or hepatic vein, a maximum of up to 45 recruited patients was allowed.

Statistics

Continuous variables are reported as means and standard deviations, and categorical variables as percentages unless otherwise noted. Correlation between the two measurement methods was assessed using scatter plots and Bland-Altman plots.[25] Pearson's correlation coefficient and 95% CIs are presented. Variances of measurements were compared using the F-test. There were no missing data. Statistics were performed using SAS® Enterprise 8.3. A two-sided p value less than 0.05 was considered statistically significant. For exploratory p values, of which there were 10, the Hochberg procedure was used to adjust for multiple testing when assessing the statistical significance of associations between clinical factors and portal hypertension. Those 10 p values included the association of EUS-PPG with esophageal varices, portal hypertensive gastropathy and thrombocytopenia, and disparity of HVP measurements with Child-Pugh score, model for end-stage liver disease (MELD) score, BMI, hepatic vein access point, depth of liver parenchyma traversed, distance between hepatic vein puncture and IVC ostium, and diameter of the target segment of the hepatic vein.

Results

Study population and procedure

From a total of 35 patients, three were excluded because of hemodynamic instability at the time of the pressure measurements, 10 because of intrahepatic shunts that could not be avoided during HVPG measurements and one because of inadequate HVPG tracings determined during independent review. Therefore, 21 patients were included in the final analysis (Fig. S1): 13 patients from Leuven and 8 patients from Barcelona. Patients were mostly men (81.0%), with a mean age of 62.3 ± 8.7 years. All patients had underlying cirrhosis, which was predominantly alcohol-related (57.1%). One patient had previously undergone a liver transplant. The mean MELD score was 10.8 ± 4.2 and most patients had Child-Pugh class B cirrhosis. Table 1 shows all baseline patient characteristics.

During the EUS procedure, the upper gastrointestinal tract was endoscopically evaluated for signs of portal hypertension. Portal hypertensive gastropathy was seen in 38.1% of patients and 66.7% had (gastro)esophageal varices. All EUS-guided punctures were performed via the transgastric route. The vein targeted via EUS was either the left (11/21) or middle (10/21) hepatic vein and either the umbilical portion of the left branch (13/21) or main portion (8/21) of the intrahepatic portal vein. Table 2 presents details of the EUS procedures.

Transjugular and EUS-guided pressures

All patients underwent simultaneous transjugular HVPG and EUS-guided PPG measurements under general anesthesia. Results for mean HVPG and EUS-PPG under general anesthesia were similar (11.9 ± 5.2 mmHg and 10.9 ± 5.6 mmHg, respectively, $p = 0.2332$) and showed a good correlation of 0.74 (p = 0.0001, Fig. 1A). When comparing the individual

Table 1. Patient demographics and characteristics.

**Baseline patient demographics and characteristics**	
Age (years)	62.3 ± 8.7
Male sex (%)	81.0%
BMI (kg/m^2)	26.0 ± 6.1
Cirrhosis (based on clinical, imaging and/or pathological findings)	100%
Etiology of liver disease	
Alcohol-related	57.1%
MASLD	19.0%
Autoimmune hepatitis	4.8%
Cryptogenic	4.8%
Combination	14.3%
Previous episodes of portal hypertension-related bleeding	19.1%
Ascites	
None	33.3%
Grade 1 (minimal)	0%
Grade 2 (moderate)	19.0%
Grade 3 (severe)	47.6%
Hepatic encephalopathy	
None	100%
Grade I – II	0%
Grade III – IV	0%
NSBB use prior to study procedure	52.4%
**Baseline lab values**	
Total serum bilirubin (mg/dl)	1.5 ± 0.9
Serum albumin (g/L)	34.6 ± 7.2
Platelet count (10^9/L)	160.2 ± 74.9
Serum creatinine (mg/dl)	1.1 ± 0.3
eGFR (ml/min)	80.4 ± 20.7
INR	1.2 ± 0.1
Serum sodium (mmol/L)	137.2 ± 4.1
**Non-invasive tests**	
Child-Pugh score	7.4 ± 1.9
Class A	33.3%
Class B	57.2%
Class C	9.5%
MELD score	10.8 ± 4.2
Liver elastography (kPa) (n = 15)	38.1 ± 22.3
**Referral procedure**	
HVPG ± transjugular liver biopsy	28.6 % (6/21)
TIPS	71.4 % (15/21)

Numbers represent means ± SD, and percentages.
eGFR, estimated glomerular filtration rate; HVPG, hepatic venous pressure gradient; INR, international normalized ratio; MASLD, metabolic syndrome associated steatotic liver disease; MELD, model of end-stage liver disease; NSBB, non-selective beta-blocker; TIPS, transjugular portosystemic pressure gradient.

Table 2. EUS procedure information.

**Endoscopic assessment**	
Varices	66.7%
Esophageal varices	52.4%
GOV type 1	14.3%
GOV type 2	0%
IGV type 1 or 2	0%
Portal hypertensive gastropathy	38.1%
Mild-moderate	23.8%
Severe	14.3%
**Hepatic vein GI access point (EUS)**	
Transgastric	100%
Transduodenal	0%
Left hepatic vein	52.4%
Middle hepatic vein	47.6%
Right hepatic vein	0%
**Hepatic vein assessment (EUS)**	
Depth of liver parenchyma traversed (mm)	31.1 ± 5.2
Distance between hepatic vein puncture site and IVC ostium (mm)	18.1 ± 10.1
Diameter of the target segment of the hepatic vein (mm)	6.0 ± 6.6
**Portal vein GI access point (EUS)**	
Transgastric	100%
Transduodenal	0%
Left portal vein branch	61.9%
Right portal vein branch	0%
Main portal vein	38.1%
**Portal vein assessment (EUS)**	
Depth of liver parenchyma traversed (mm)	33.6 ± 14.2
Diameter of the target segment of the portal vein (mm)	9.5 ± 10.5

Numbers represent means ± SD, and percentages.
EUS, endoscopic ultrasound; GI, gastrointestinal; GOV, gastroesophageal varices; IGV, isolated gastric varices; IVC, inferior vena cava.

components of the gradients, the correlation of the portal pressures (WHVP *vs.* EUS-PVP) showed an even higher correlation of 0.85 (p <0.0001, Fig. 1C), while the hepatic pressure (FHVP *vs.* EUS-HVP) correlation was slightly lower (0.72, p = 0.0003, Fig. 1E). Table 3 shows the mean transjugular and EUS-guided pressures, while Table 4 summarizes the correlation for the paired pressures. Bland-Altman plots illustrating the agreement of the individual pressure measurements are shown (Fig. 1B,D,F). Notably, a higher discrepancy was seen between the EUS-HVP and transjugular FHVP when pressures increased, as depicted by the regression line in the Bland-Altman plot included in Fig. 1F. The EUS-guided portal vein and hepatic pressures did not differ based on the location of the pressure measurements (left *vs.* main portal vein, left *vs.* middle hepatic vein; Table S3).

In the 15 patients undergoing TIPS, an excellent correlation of 0.91 (p <0.0001, Fig. 2A) was observed when direct portal pressures (measured by insertion of a catheter into the portal vein) were compared to EUS-guided portal pressure measurement. In these 15 patients, the correlation between the direct transjugular PVP and WHVP was comparable, albeit slightly lower (0.89 *vs.* 0.91, Fig. S2).

To assess the effect of general anesthesia, HVPG measurements were first performed without sedation and repeated after induction of general anesthesia with propofol. The HVPG decreased under general anesthesia from 14.2 ± 5.1 mmHg to 11.9 ± 5.2 mmHg (p <0.0001). On average, the WHVP pressure decreased (22.9 ± 6.3 mmHg to 21.3 ± 6.4 mmHg, p = 0.0074) while the FHVP slightly, but not significantly, increased (8.7 ± 3.4 mmHg to 9.3 ± 2.9 mmHg, p = 0.1742).

Examining the clinically relevant HVPG cut-offs of 10 mmHg, which defines CSPH and the threshold for varices formation and hepatic decompensation, and 16 mmHg, linked to increased mortality in patients with cirrhosis, reveals slight variations in patient classification depending on anesthesia and measurement technique.[2,3] Using HVPG measurements under local anesthesia (standard of care), 81.0% of patients were identified as having CSPH (10 mmHg threshold), while this decreased to 76.2% for HVPG under general anesthesia and 66.7% with EUS-PPG. Similarly, at the 16 mmHg cut-off, 42.9% of patients exceeded this cut-off using HVPG under local anesthesia, compared to 14.3% for both HVPG and EUS-PPG under general anesthesia. Table 5 summarizes these results.

Fig. S3 compares the pressure correlations between the included patients and the patients who were excluded because of intrahepatic veno-venous shunts that could not be avoided during HVPG measurements.

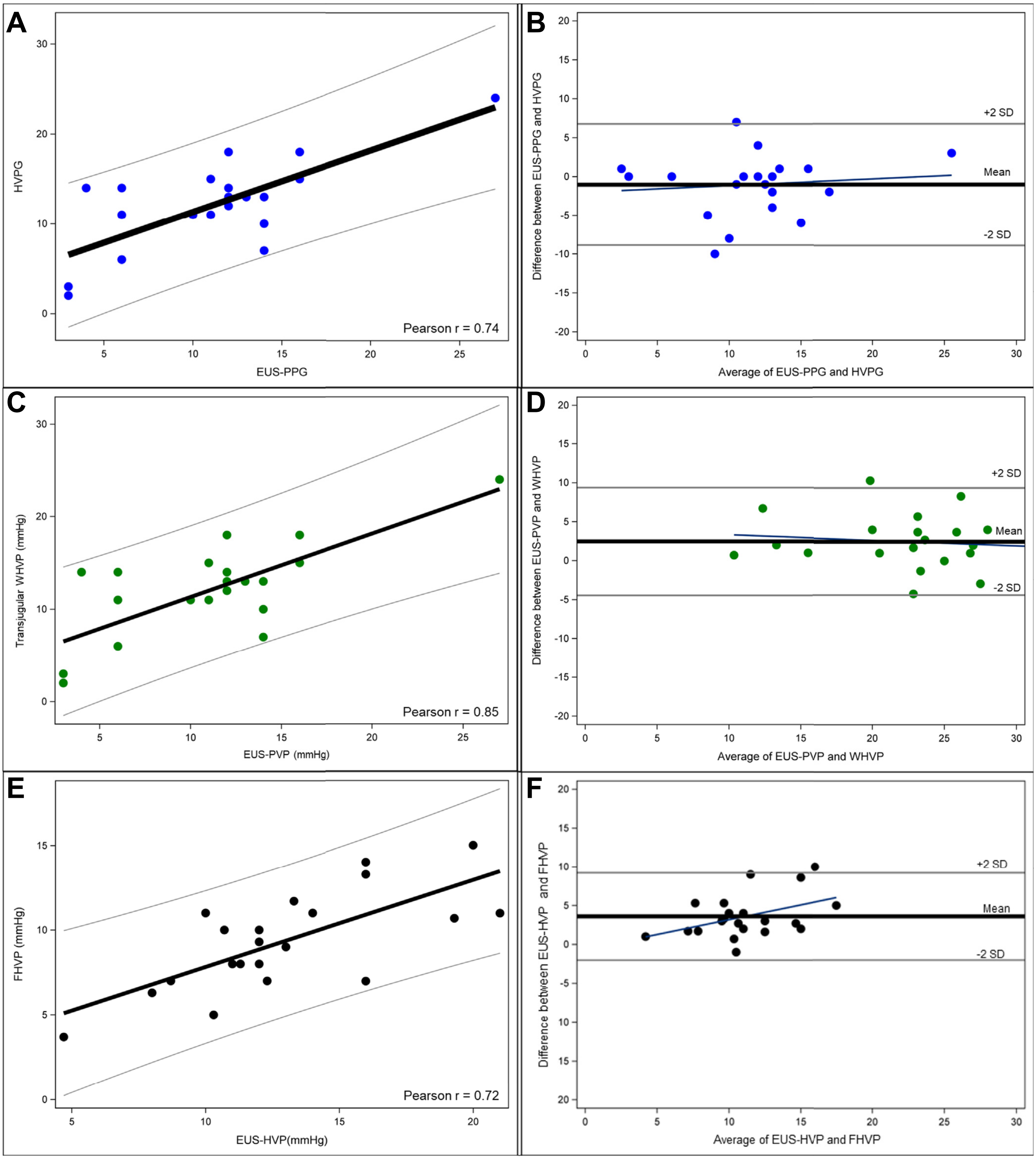

Fig. 1. Correlation and Bland-Altman plots of the pressure gradients and individual pressures using the transjugular and EUS-guided approach (all under general anesthesia). (A) Correlation of pressure gradients (transjugular HVPG *vs.* EUS-PPG), Pearson r = 0.74, *p* = 0.0001; (B) Bland-Altman plot for pressure gradients, (C) Correlation of portal vein pressures (transjugular WHVP *vs.* EUS-PVP), Pearson r = 0.85, *p* <0.0001; (D) Bland-Altman plot for portal vein pressures, (E) Correlation of hepatic vein pressures (transjugular FHVP *vs*. EUS-HVP), Pearson r = 0.72, *p* <0.0003; (F) Bland-Altman plot for hepatic vein pressures. EUS, endoscopic ultrasound; FHVP, free hepatic venous pressure; HVP, hepatic venous pressure; HVPG, hepatic venous pressure gradient; PPG, portal pressure gradient; PVP, portal venous pressure; TIPS, transjugular intrahepatic portosystemic shunt; WHVP, wedged hepatic venous pressure.

Table 3. Transjugular and EUS-guided pressure measurements.

Transjugular and EUS-guided pressure measurements	mmHg
**No/mild sedation**	
Transjugular HVPG	14.2 ± 5.1
WHVP	22.9 ± 6.3
FHVP	8.7 ± 3.4
**General anesthesia**	
Transjugular HVPG	11.9 ± 5.2
WHVP	21.3 ± 6.4
FHVP	9.3 ± 2.9
Transjugular direct portal venous pressure (patients undergoing TIPS)	22.9 ± 5.3
EUS-guided PPG	10.9 ± 5.6
PVP	23.8 ± 6.0
HVP	12.9 ± 4.0

Numbers represent means ± SD, and percentages.
HVPG, hepatic venous pressure gradient; WHVP, wedged hepatic venous pressure; FHVP, free hepatic venous pressure; TIPS, transjugular intrahepatic portosystemic shunt; EUS, endoscopic ultrasound; PPG, portal pressure gradient; PVP, portal venous pressure; HVP, hepatic venous pressure.

Association with clinical and lab-based features of portal hypertension

Patients with esophageal varices had significantly higher EUS-PPG values compared to patients without varices (13.5 ± 4.6 [n = 11] *vs.* 5.6 ± 2.8 mmHg [n = 10], p <0.001). Although not statistically significant (after correction using the Hochberg procedure), elevated EUS-PPG values were also found in patients with portal hypertensive gastropathy (13.9 ± 6.0 [n = 8] *vs.* 9.0 ± 4.5 [n = 13], p = 0.05) and patients with thrombocytopenia (11.6 ± 7.1 [n = 10] *vs.* 10.0 ± 3.4 [n = 11], p = 0.51). Liver elastography was available in 15 patients, demonstrating a moderate correlation with EUS-PPG (r = 0.52, p = 0.05).

Technical success and adverse events

Three measurements of HVP and PVP were obtained in all patients using the EchoTip® Insight™ system; thus, the technical success rate was 100%.

All patients underwent a 30-day post-procedure follow-up. Twelve patients experienced adverse events, of which only one (4.8%) was regarded as procedure-related (Table 6): a laceration of the tunica intima of the portal vein during transjugular access for TIPS placement. As a result, a second transhepatic puncture was performed, successfully achieving TIPS access. No adverse events were directly related to the EUS-guided PPG and no device deficiencies occurred. Of note, all hepatic encephalopathy events were in patients who underwent TIPS during the study. The single patient who experienced bleeding during follow-up presented with melena and was diagnosed with portal hypertension-related colopathy on endoscopy.

Subgroup analyses

A *post hoc* subgroup analysis was conducted to investigate factors potentially contributing to discrepancies between the EUS-HVP and transjugular FVHP. As previously mentioned, a

Table 4. Correlation of pressure measurements.

	Pearson correlation	*p* value
Primary endpoint		
HVPG – EUS-PPG (general anesthesia)	0.74	0.0001
Secondary endpoints		
WHVP – EUS-PVP (general anesthesia)	0.85	<0.0001
FHVP – EUS-HVP (general anesthesia)	0.72	0.0003
HVPG (local anesthesia) – HVPG (general anesthesia)	0.94	<0.0001
TIPS patients (n = 15):		
Transjugular direct portal venous pressure – EUS-PVP	0.91	<0.0001

EUS, endoscopic ultrasound; FHVP, free hepatic venous pressure; HVP, hepatic venous pressure; HVPG, hepatic venous pressure gradient; PPG, portal pressure gradient; PVP, portal venous pressure; TIPS, transjugular intrahepatic portosystemic shunt; WHVP, wedged hepatic venous pressure.

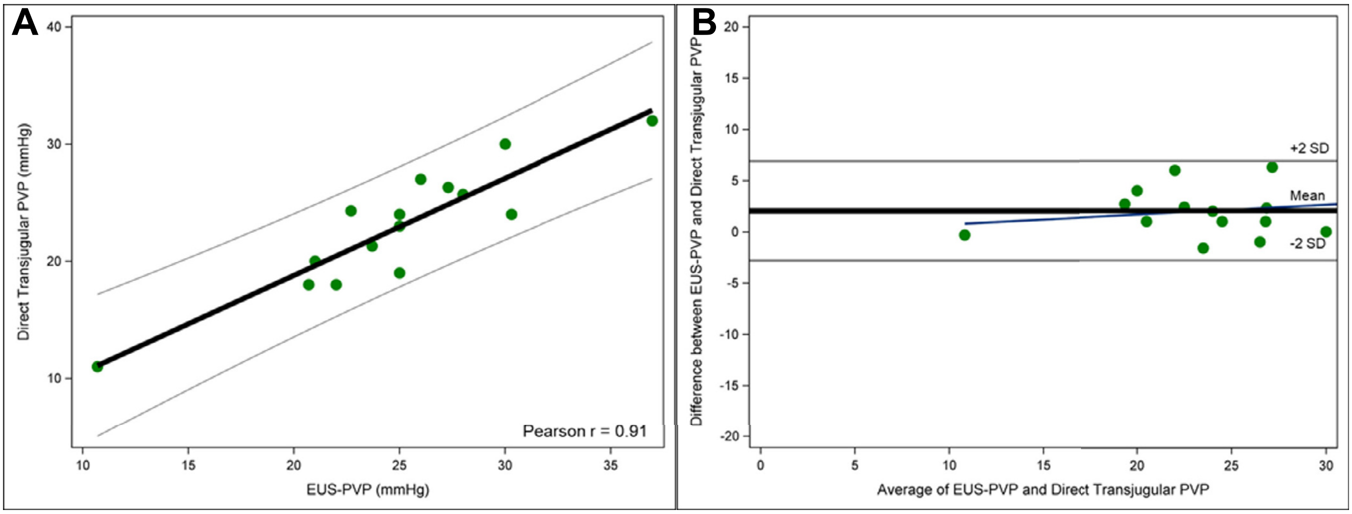

Fig. 2. Comparison of the direct transjugular portal pressure *vs.* EUS-guided portal vein pressure in patients receiving TIPS. (A) Correlation, Pearson r = 0.91, p <0.001, (B) Bland-Altman plot. EUS, endoscopic ultrasound; TIPS, transjugular intrahepatic portosystemic shunt.

Table 5. Patient classification based on clinically relevant thresholds of 10 mmHg and 16 mmHg.

Pressure threshold	≥10 mmHg	≥16 mmHg
HVPG (local anesthesia)	17/21 (81.0%)	9/21 (42.9%)
HVPG (general anesthesia)	16/21 (76.2%)	3/21 (14.3%)
EUS-PPG (general anesthesia)	14/21 (66.7%)	3/21 (14.3%)

Numbers represent means ± SD, and percentages.
EUS, endoscopic ultrasound; HVPG, hepatic venous pressure gradient; PPG, portal pressure gradient.

Table 6. Adverse events within 30 days after the EUS-PPG and HVPG procedures.

Adverse events	
Patients experiencing adverse events*	57.1% (12/21)
Hypotension	8/21
Congestive heart failure	1/21
Hepatic encephalopathy (post-TIPS)	4/21
Infection	5/21
Thrombosis (lower extremity)	1/21
Vascular injury	1/21
Edema	2/21
Portal hypertension-related bleeding	1/21
Acute kidney injury	1/21
Thrombocytopenia needing transfusion	1/21
Anemia needing transfusion	1/21
Adverse events related to the study procedure	4.8% (1/21)
Adverse events due to a device deficiency	0%
Adverse events leading to death	0%

Numbers represent proportions and percentages.
EUS, endoscopic ultrasound; PPG, portal pressure gradient; TIPS, transjugular intrahepatic portosystemic shunt.
*Some patients experienced more than one adverse event.

higher discrepancy was seen at higher pressures (Fig. 1F). Results showed a trend towards higher Child-Pugh score and shorter distance between the hepatic vein puncture site and IVC ostium in patients with a discrepancy of 3 mmHg or more between EUS-HVP and FHVP (Table S4).

Since only six patients with MASLD (±alcohol-related etiology) were included, the sample size was considered too small to perform a subgroup analysis on the influence of etiologies with a potential presinusoidal component of portal hypertension.

Discussion

The progression to CSPH marks a pivotal turning point in the natural history of cirrhosis. At this juncture, patients are at heightened risk for severe complications, including ascites, varices, hepatocellular carcinoma, and acute decompensating events such as variceal bleeding and bacterial infections.[2] These complications significantly influence the clinical trajectory and prognosis of patients with cirrhosis. In recent years, there has been a concerted effort to identify CSPH before the manifestation of these complications. Early intervention aimed at reducing portal pressure has the potential to prevent or delay hepatic decompensation.[26] CSPH is traditionally diagnosed by measuring the HVPG, with a gradient of ≥10 mmHg indicating clinical significance. However, HVPG measurement is invasive, limited in availability, and may be less accurate in case of presinusoidal causes of portal hypertension and MASLD.[2,3] Non-invasive methods, such as liver elastography alone or in combination with platelet count, have been proposed as alternatives to infer elevated portal pressure.[3] While these methods offer practical advantages, they do not yet cover the full spectrum and leave a 'grey zone', highlighting the need for improved non-invasive tools or alternative methods to measure portal pressure.[27,28] Furthermore, liver elastography can be influenced by factors such as steatosis, hepatic inflammation, congestion and cholestasis, potentially leading to overestimation or underestimation of portal hypertension.[29] Moreover, after initiating non-selective beta-blocker (NSBB) therapy, routine measurement of portal pressure is not commonly recommended. This is despite evidence suggesting that up to 50% of patients may exhibit inadequate responses to NSBBs, underscoring the need for individualized assessment and monitoring.[30]

The ENCOUNTER study demonstrates that an EUS-guided strategy for measuring portal pressure is comparable to the current gold standard, HVPG, when measured simultaneously in the same patients and under the same conditions. Additionally, an excellent correlation was observed between EUS-guided and direct transjugular portal pressure measurements in patients undergoing TIPS, which represents the most accurate assessment method. The procedure was technically successful in all cases, with no adverse events related to the EUS-guided pressure measurements observed when performed under general anesthesia by experienced operators.

To our knowledge, only two studies have previously reported the correlation between HVPG and EUS-PPG. One recently published prospective study by Martinez-Moreno *et al.*[31] showed good correlation between standard-of-care HVPG and EUS-PPG under propofol anesthesia in patients with cirrhosis (r = 0.82, n = 30), even though measurements were not performed simultaneously as in the current study. Another study by Zhang *et al.*[18] also showed an excellent correlation in patients with (sub)acute portal hypertension (r = 0.92, n = 9). These studies, as well as others reporting the correlation of EUS-PPG with fibrosis and/or non-invasive markers of liver disease, have similarly reported high technical success rates (91.7-100%) and low adverse event rates (0-9.6%).[17,18,21–23,31]

Some limitations should be acknowledged. First, a relatively large number of patients had to be excluded due to hemodynamic instability at the time of the pressure measurements and/or the presence of intrahepatic shunts that interfered with HVPG measurement, which made the pressure readings unreliable. According to the literature these shunts can be found in up to one-third of patients with end-stage liver disease.[10–13] This hemodynamic instability can be attributed to the use of general anesthesia and the inclusion of patients with more advanced decompensated cirrhosis, necessitating the use of short-acting vasoactive drugs during the procedure.[10] Secondly, for simultaneous HVPG and EUS-PPG measurement to be performed safely and under complete control, the use of general anesthesia was mandated by the central review board. The administration of propofol during EUS-PPG measurement has been a topic of debate. Propofol sedation can influence pressure measurements and typically results in lower WHVP and, consequently, lower HVPG values.[32,33] HVPG was assessed under local anesthesia first (as standard of care) in each patient before propofol administration in this study, where we confirmed the aforementioned effect of propofol. The simultaneous nature of the measurements meant that the

use of propofol affected both EUS-guided and transjugular pressure results equally. Nevertheless, it is important to note, as shown in this study, that the use of general anesthesia can lead to an underestimation of portal hypertension severity and potential misclassifications when predefined thresholds, such as 10 and 16 mmHg, are used. Therefore, for PPG measurements performed outside of a research context, sedatives such as midazolam may be preferred, as midazolam will not affect PPG values at low doses.[34] Notably, Martinez-Moreno *et al.*[28] reported a strong correlation between EUS-PPG and HVPG, even when measured in separate sessions, using propofol sedation for EUS-PPG and no sedation or low-dose midazolam for HVPG. Future studies should explore the feasibility of performing EUS-PPG measurements under low-dose midazolam. Thirdly, while slight discrepancies between EUS-PVP and WHVP can be attributed to the difference between direct and indirect measurement techniques, the cause of inconsistencies between EUS-HVP and transjugular FHVP is uncertain. A key strength of this study is its design, which enabled simultaneous EUS-guided and transjugular measurements in the same patients. This reduces the possibility that differences in pressure measurements were due to factors such as propofol sedation, large-volume paracentesis, crystalloid infusions, positive pressure ventilation, *etc*. The procedures were performed by experienced endoscopists maintaining a relaxed scope position and avoiding gastric insufflation whenever possible. Still, our *post hoc* analysis showed a possible trend towards more discrepancy in the hepatic pressures when a hepatic vein puncture site closer to the IVC was chosen, which coincides with a more angulated position in the stomach, possibly requiring more scope and elevator tension. We also observed increased variability in hepatic vein measurements at higher pressures, along with a trend toward higher Child-Pugh scores, indicating a possible association with the severity of liver disease. However, MELD scores remained similar. The limited size of our study cohort makes it difficult to establish with certainty the factors influencing these pressure differences. Unfortunately, no other authors have reported on the correlation of the individual pressures contributing to the gradients. In our experience, the EUS-guided HVP is often, but not always, slightly higher than the transjugular FHVP (Fig. 1F). Notably, in the few studies also reporting mean HVP, relatively high pressures were observed as well, namely a mean HVP of 15.9 ± 3.5 mmHg (n = 30, compared to a mean FHVP of 12.7 ± 5.4 mmHg) in the study by Martinez-Moreno *et al.*,[31] an HVP of 15.9 ± 4.3 mmHg (n = 24) in the study by Hajifathalian *et al.*[19] and an HVP of 15.8 ± 3.4 mmHg (n = 12) in the study by Lesmana *et al.*[35] Overestimating HVP could again result in a relevant underestimation of the pressure gradient, particularly when values fall below established thresholds, thereby impacting clinical decision-making. This raises the challenging question of whether assessing direct portal pressure alone, rather than relying on a calculated gradient, might suffice for evaluating disease severity and prognosis. Fourth, the ENCOUNTER study intentionally included patients undergoing TIPS placement, as portal pressure is measured directly via transjugular access in these cases, allowing direct comparison with EUS-guided portal pressure measurements. However, patients with decompensated liver disease are not the patient population expected to be referred for EUS-PPG in clinical practice. Outside the scope of this study, transjugular portal pressure measurement may be more appropriate for end-stage liver disease, especially in the presence of coagulopathy or large-volume ascites. However, it should be mentioned that, even in this population with higher perceived periprocedural risk, no bleeding events or cases of spontaneous/secondary bacterial peritonitis were observed following EUS-guided pressure measurement.

The primary application of EUS-PPG will likely be most relevant in three distinct patient groups: (a) patients with chronic liver disease who have conflicting non-invasive test results, (b) patients in whom HVPG is known to underestimate true portal pressure, (c) patients requiring additional endoscopic procedures. First, EUS-PPG may be particularly beneficial for patients with advanced chronic liver disease in whom portal hypertension is suspected but not yet clinically evident, and non-invasive measures have failed to guide clinical decision-making. This is particularly relevant in patients with MASLD, where hepatic steatosis and obesity can interfere with the accuracy of liver and spleen elastography, and HVPG may underestimate portal hypertension due to a presinusoidal component. The potential of EUS-PPG as a direct measurement tool for portal hypertension in MASLD warrants further investigation. Second, EUS-PPG is a valuable direct alternative in cases where HVPG is known to underestimate portal pressure, such as in patients with presinusoidal portal hypertension or patients with intrahepatic veno-venous shunts that cannot be bypassed. Additional research is needed in these specific patient populations. Third, the use of the EUS strategy for portal pressure measurement is particularly advantageous as it allows for a 'one-stop procedure'. This integrated endoscopic and endosonographic approach offers clinicians valuable insights for diagnosing and stratifying liver disease. It enables the evaluation of liver-related complications, assessment of histopathologic inflammation and fibrosis through biopsy and/or elastography, and measurement of portal pressure, all while minimizing patient burden by eliminating the need for separate procedures. Future studies should evaluate the cost-effectiveness of the 'one-stop procedure'. While the ENCOUNTER study represents in important next step in evaluating the efficacy of EUS-PPG as a new measure for portal pressure, additional research is needed before it can be widely implemented in clinical practice. Key areas for further investigation include the feasibility of EUS-PPG under low-dose midazolam in the left lateral position and the use of EUS-PPG to assess response to NSBB treatment.

In conclusion, EUS-PPG seems to be a valid and safe alternative to HVPG for assessing the presence and severity of portal hypertension. This study shows that portal pressure can be assessed with high accuracy via EUS-guided puncture. However, attention must be paid to technical challenges, including the potential overestimation of HVP and the influence of general anesthesia. EUS-PPG is particularly attractive for patients with chronic liver disease who have conflicting non-invasive test results, require additional endoscopic procedures or in cases where HVPG is known to underestimate true portal pressure.

Affiliations

[1]Department of Gastroenterology and Hepatology, University Hospitals Leuven, KU Leuven, Leuven, Belgium; [2]Department of Chronic Diseases, Metabolism and Aging (CHROMETA), Catholic University of Leuven, Leuven, Belgium; [3]Department of Internal Medicine B, University of Münster, Münster, Germany; [4]Department of Radiology, Interventional Radiology, University Hospital Leuven, Leuven, Belgium; [5]MED-Surg Division, Cook Medical, Bloomington, Indiana, USA; [6]Cook Research Incorporated, West Lafayette, Indiana, USA; [7]Department of Anesthesiology, Critical Care and Pain Medicine, Hospital Clinic de Barcelona, IDIBAPS, Barcelona, Spain; [8]Endoscopy Unit, Department of Digestive Diseases, Parc Tauli University Hospital, Investigation and Innovation Institute Parc Tauli I3PT, Universitat Autonoma of Barcelona, Sabadell, Spain; [9]Endoscopy Unit, Service of Gastroenterology, Institut Clinic de Malalties Digestives i Metaboliques, Hospital Clinic, IDIBAPS, CIBERehd, University of Barcelona, Barcelona, Spain; [10]Barcelona Hepatic Hemodynamic Laboratory, Liver Unit, Hospital Clinic, IDIBAPS, Department de Medicina I Ciències de la Salut. Universitat de Barcelona, Barcelona. Health Care Provider of the European Reference Network on Rare Liver Disorders (ERN-Rare Liver), Spain; [11]Department of Gastroenterology and Hepatology, Erasmus MC University Medical Center, Rotterdam, the Netherlands

Abbreviations

CSPH, clinically significant portal hypertension; EUS, endoscopic ultrasound; FHVP, free hepatic venous pressure; HVP, hepatic venous pressure; HVPG, hepatic venous pressure gradient; IVC, inferior vena cava; MASLD, metabolic syndrome associated steatotic liver disease; MELD, model of end-stage liver disease; NSBBs, non-selective beta-blockers; PPG, portal pressure gradient; PVP, portal venous pressure; SBP, spontaneous bacterial peritonitis; TIPS, transjugular intrahepatic portosystemic shunt; WHVP, wedged hepatic venous pressure.

Financial support

This project was funded by Cook Medical. Employees of Cook Medical collaborated with the investigators on study design, data acquisition, analysis, interpretation of data, and report writing.

Conflict of interest

WL has consultancy agreements with Pentax Medical, Boston Scientific and CSL Behring. AG has been awarded a grant from Cook Medical. AB has received speaking fees from Gore. MB serves as a consultant for Boston Scientific, Cook Medical, Pentax Medical, EcoLab and Interscope and has received grants for industry and investigator-initiated research from Boston Scientific, Cook Medical, Pentax Medical, InterScope and Mylan. JCGP serves as an advisor for GSK, has received speaker fees from Gore and Cook Medical, and has been awarded grants from Mallinckrodt, Cook Medical, and AstraZeneca. SV holds the Boston Scientific Chair in Interventional Endoscopy as well as the Cook chair for the study of portal pressure measurement, and holds consultancy agreements with Cook, Pentax and Olympus. All other authors declare no conflicts of interest relevant to this work.

Please refer to the accompanying ICMJE disclosure forms for further details.

Authors' contributions

Conceptualization and methodology: SV, WL, JCGP, MB and DW designed the research approach and developed the methodologies. Investigation: LB, GM, AC, OS, AG, AB, JCGP, WL and SV conducted the. Experimental investigations and data collection. Formal analysis, data curation and visualization: EV and CY were responsible for data analysis, data management, and graphical representation of data. Writing – original draft: EV drafted the initial manuscript. Resources, Funding and Project Administration: Cook Research Incorporated provided resources, funding and managed the project administration. Supervision: JCGP, WL and SV oversaw the research project, providing strategic guidance and leadership. Manuscript Review & Editing: All authors participated in reviewing and editing the manuscript, providing critical feedback and approval of the final version.

Data availability statement

Data underlying the results reported in this article are available upon reasonable request from the corresponding author, immediately after publication and ending 5 years after publication, subject to review and approval by the study sponsor. Interested researchers may review the "Cook Research Incorporated Policy on Access to Clinical Study Data" at https://www.cookresearchinc.com/extranet/data-access.html and submit a complete research proposal to request data access.

Acknowledgements

We would like to express our heartfelt gratitude to the (study) nurses from the hepatology, endoscopy, and interventional radiology teams at the participating sites, including Petra Windmolders, Tine Vrancken, Natalie Van den Ende, Joana Codina and Lara Orts, for their time and dedication in successfully executing this study. We extend our thanks to the team at Cook Medical and Cook Research Incorporated, particularly Nicholas D. Dey, for his contributions to medical writing, including data review, summary and editorial assistance, and Simon Brouwers for his invaluable support in performing all procedures. We extend our appreciation to Prof. Juan Abraldes, who served as an independent reviewer for the HVPG tracings in this study. Cook Research Incorporated also wishes to acknowledge the contributions of Dr. Kenneth J. Chang and Dr. William Brugge. We would like to acknowledge Prof Rob de Man for his valuable contributions to the conceptualization of the study. Lastly, we would like to honor the contributions of Dr. Adriaan Moelker, who sadly passed away during the course of this study.

Supplementary data

Supplementary data to this article can be found online at https://doi.org/10.1016/j.jhepr.2025.101466.

References

[1] Devarbhavi H, Asrani SK, Arab JP, et al. Global burden of liver disease: 2023 update. J Hepatol 2023;79(2):516–537. https://doi.org/10.1016/j.jhep.2023.03.017.

[2] Berzigotti A, Seijo S, Reverter E, et al. Assessing portal hypertension in liver diseases. Expert Rev Gastroenterol Hepatol 2013;7(2):141–155. https://doi.org/10.1586/egh.12.83.

[3] De Franchis R, Bosch J, Garcia-Tsao G, et al. Baveno VII – renewing consensus in portal hypertension. J Hepatol 2022;76(4):959–974. https://doi.org/10.1016/j.jhep.2021.12.022.

[4] Groszmann RJ, Wongcharatrawee S. The hepatic venous pressure gradient: anything worth doing should Be done right. Hepatology 2004;39(2):280–283. https://doi.org/10.1002/hep.20062.

[5] Gunarathne LS, Rajapaksha H, Shackel N, Angus PW, Herath CB. Cirrhotic portal hypertension: from pathophysiology to novel therapeutics. World J Gastroenterol 2020;26(40):6111–6140. https://doi.org/10.3748/wjg.v26.i40.6111.

[6] Ripoll C, Groszmann R, Garcia-Tsao G, et al. Hepatic venous pressure gradient predicts clinical decompensation in patients with compensated cirrhosis. Gastroenterology 2007;133(2):481–488. https://doi.org/10.1053/j.gastro.2007.05.024.

[7] D'Amico G, Garcia-Pagan JC, Luca A, Bosch J. Hepatic vein pressure gradient reduction and prevention of variceal bleeding in cirrhosis: a systematic review. Gastroenterology 2006;131(5):1611–1624. https://doi.org/10.1053/j.gastro.2006.09.013.

[8] Vorobioff J, Groszmann RJ, Picabea E, et al. Prognostic value of hepatic venous pressure gradient measurements in alcoholic cirrhosis: a 10-year prospective study. Gastroenterology 1996;111:701–709.

[9] Ferrusquía-Acosta J, Bassegoda O, Turco L, et al. Agreement between wedged hepatic venous pressure and portal pressure in non-alcoholic steatohepatitis-related cirrhosis. J Hepatol 2021;74(4):811–818. https://doi.org/10.1016/j.jhep.2020.10.003.

[10] Ma J, Gong X, Luo J, et al. Impact of intrahepatic venovenous shunt on hepatic venous pressure gradient measurement. J Vasc Interv Radiol 2020;31(12):2081–2088. https://doi.org/10.1016/j.jvir.2020.08.027.

[11] Wang L, Song Q, Yue Z, et al. Study on the correlation between PPG and HVPG in patients with portal hypertension. Chin J Hepatol 2022;30(7):722–727.

[12] Perelló A, Escorsell À, Bru C, et al. Wedged hepatic venous pressure adequately reflects portal pressure in hepatitis C virus-related cirrhosis. Hepatology 1999;30(6):1393–1397. https://doi.org/10.1002/hep.510300628.

[13] Osada Y, Kanazawa H, Narahara Y, et al. Wedged hepatic venous pressure does not reflect portal pressure in patients with cirrhosis and hepatic venovenous communications. Dig Dis Sci 2008;53(1):7–13. https://doi.org/10.1007/s10620-007-0039-3.

[14] Laleman W, Vanderschueren E, Mehdi ZS, et al. Endoscopic procedures in hepatology: current trends and new developments. J Hepatol September 2023. https://doi.org/10.1016/j.jhep.2023.08.032. Published online.

[15] Lai L, Poneros J, Santilli J, Brugge W. EUS-guided portal vein catheterization and pressure measurement in an animal model: a pilot study of feasibility. Gastrointest Endosc 2004;59(2):280–283.

[16] Fujii-Lau LL, Leise MD, Kamath PS, Gleeson FC, Levy MJ. Endoscopic ultrasound-guided portal-systemic pressure gradient measurement. Endoscopy 2014;46:E654–E656. https://doi.org/10.1055/s-0034-1390845.

[17] Huang JY, Samarasena JB, Tsujino T, et al. EUS-guided portal pressure gradient measurement with a simple novel device: a human pilot study. Gastrointest Endosc 2017;85(5):996–1001. https://doi.org/10.1016/j.gie.2016.09.026.

[18] Zhang W, Peng C, Zhang S, et al. EUS-guided portal pressure gradient measurement in patients with acute or subacute portal hypertension. Gastrointest Endosc 2021;93(3):565–572. https://doi.org/10.1016/j.gie.2020.06.065.

[19] Hajifathalian K, Westerveld D, Kaplan A, et al. Simultaneous EUS-guided portosystemic pressure measurement and liver biopsy sampling correlate with clinically meaningful outcomes. Gastrointest Endosc 2022;95(4):703–710. https://doi.org/10.1016/j.gie.2021.11.037.

[20] Choi AY, Chang KJ, Samarasena JB, et al. Endoscopic ultrasound-guided porto-systemic pressure gradient measurement correlates with histological hepatic fibrosis. Dig Dis Sci 2022;67(12):5685–5692. https://doi.org/10.1007/s10620-022-07418-7.

[21] Kim S, Lewey S, Meuller L, Adler DG. Endohepatology in clinical practice: EUS-guided portal pressure measurement combined with EUS-guided liver biopsy and variceal screening and treatment in outpatients. Endosc Ultrasound 2024;13(2):89–93. https://doi.org/10.1097/eus.0000000000000030.

[22] Choi AY, Kolb J, Shah S, et al. Endoscopic ultrasound-guided portal pressure gradient with liver biopsy: 6 years of endo-hepatology in practice. J Gastroenterol Hepatol (Australia) 2022;37(7):1373–1379. https://doi.org/10.1111/jgh.15875.

[23] Kolb JM, Monachese M, Rubin RA, et al. Endoscopic ultrasound–guided portosystemic pressure gradient correlates with clinical parameters and liver histology. Clin Gastroenterol Hepatol January 2025. https://doi.org/10.1016/j.cgh.2024.12.022. Published online.

[24] Moinester M, Gottfried R. Sample size estimation for correlations with pre-specified confidence interval. Quant Method Psychol 2014;10(2):124–130.

[25] Bland JM, Altman DG. Statistical methods for assessing agreement between two methods of clinical measurement. The Lancet 1986:307–310. Published online.

[26] Villanueva C, Albillos A, Genescà J, et al. β blockers to prevent decompensation of cirrhosis in patients with clinically significant portal hypertension (PREDESCI): a randomised, double-blind, placebo-controlled, multicentre trial. The Lancet 2019;393(10181):1597–1608. https://doi.org/10.1016/S0140-6736(18)31875-0.

[27] Dajti E, Ravaioli F, Marasco G, et al. A combined Baveno VII and spleen stiffness algorithm to improve the non-invasive diagnosis of clinically significant portal hypertension in patients with compensated advanced chronic liver disease. Am J Gastroenterol 2022;117(11):1825–1833. https://doi.org/10.14309/ajg.0000000000001887.

[28] Jachs M, Odriozola A, Turon F, et al. Spleen stiffness measurement by vibration-controlled transient elastography at 100 Hz for non-invasive predicted diagnosis of clinically significant portal hypertension in patients with compensated advanced chronic liver disease: a modelling study. Lancet Gastroenterol Hepatol 2024;9(12):1111–1120. https://doi.org/10.1016/S2468-1253(24)00234-6.

[29] Dietrich CF, Bamber J, Berzigotti A, et al. EFSUMB guidelines and recommendations on the clinical use of liver ultrasound elastography, update 2017 (long version). Ultraschall in der Medizin 2017;38(4):e16–e47. https://doi.org/10.1055/s-0043-103952.

[30] Sinagra E, Perricone G, D'Amico M, Tinè F, D'Amico G. Systematic review with meta-analysis: the haemodynamic effects of carvedilol compared with propranolol for portal hypertension in cirrhosis. Aliment Pharmacol Ther 2014;39(6):557–568. https://doi.org/10.1111/apt.12634.

[31] Martinez-Moreno B, Martínez Martínez J, Herrera I, et al. Correlation of EUS-guided portal pressure gradient measurements with hepatic venous pressure gradient: a prospective study. Endoscopy 2024. https://doi.org/10.1055/a-2369-0759. Published online July 18.

[32] Reverter E, Blasi A, Abraldes JG, et al. Impact of deep sedation on the accuracy of hepatic and portal venous pressure measurements in patients with cirrhosis. Liver Int 2014;34(1):16–25. https://doi.org/10.1111/liv.12229.

[33] Ebrahimi F, Semela D, Heim M. Impact of propofol sedation on the diagnostic accuracy of hepatic venous pressure gradient measurements in patients with cirrhosis. Hepatol Int 2022;16(4):817–823. https://doi.org/10.1007/s12072-021-10261-z.

[34] Steinlauf AF, Garcia-Tsao G, Zakko MF, et al. Low-dose midazolam sedation: an option for patients undergoing serial hepatic venous pressure measurements. Hepatology 1999;29(4):1070–1073. https://doi.org/10.1002/hep.510290421.

[35] Lesmana CRA. Technique innovation of endoscopic ultrasound portal pressure gradient measurement using standard manometer set for portal hypertension assessment. Clin Case Rep 2022;10(12). https://doi.org/10.1002/ccr3.6658.

Keywords: portal hypertension; portal pressure; endosonography; endoscopy; HVPG.
Received 27 January 2025; received in revised form 16 May 2025; accepted 20 May 2025; Available online 29 May 2025

Journal of Hepatology, Volume 7

Supplemental information

Endoscopic ultrasound-guided portosystemic pressure gradient measurement *vs.* transjugular balloon occlusion measurement in patients with cirrhosis (ENCOUNTER): A bicentric EU study

Emma Vanderschueren, Wim Laleman, Lawrence Bonne, Geert Maleux, David R. Wagner, Chyon Yeh, Andrea Calvo, Oriol Sendino, Angels Gines, Anna Baiges, Marco J. Bruno, Juan Carlos Garcia-Pagan, and Schalk van der Merwe

Endoscopic ultrasound-guided portosystemic pressure gradient measurement *vs.* transjugular balloon occlusion measurement in patients with cirrhosis (ENCOUNTER): A bicentric EU study

Emma Vanderschueren, Wim Laleman, Lawrence Bonne, Geert Maleux, David R. Wagner, Chyon Yeh, Andrea Calvo, Oriol Sendino, Angels Gines, Anna Baiges, Marco J. Bruno, Juan Carlos Garcia-Pagan, Schalk van der Merwe

Table of contents

Table S1…………………………………………………………………………………..2

Table S2…………………………………………………………………………………..3

Table S3…………………………………………………………………………………..4

Table S4…………………………………………………………………………………..4

Fig. S1……………………………………………………………………………………5

Fig. S2……………………………………………………………………………………5

Fig. S3……………………………………………………………………………………6

Table S1. Study exclusion criteria

**General criteria**	Patient is < 18 or > 85 years of age
	Patient is pregnant, breast-feeding, or planning to become pregnant during the course of the study
	Patient is unwilling or unable to sign and date the informed consent
	Patient is unwilling to comply with the follow-up study schedule
	Patients for whom endoscopic procedures are contraindicated
	Patients for whom propofol general anesthesia is contraindicated
**Medical criteria**	Platelet count <50,000 per mm3
	INR > 1.7
	eGFR < 50 mL/min/1.73m2
	Previous transjugular intrahepatic or surgical portosystemic shunt
	Previous total or partial splenectomy
	Non-cirrhotic portal hypertension
	SBP within the last three months irrespective of treatment for SBP
	Patients with known infection which is not controlled by medical intervention
	Portopulmonary hypertension
	Cardiac decompensation
	Pre-sinusoidal liver disease
	Cholestatic liver disease
	Patient who received endoscopic treatment for upper gastrointestinal variceal bleeding within the past 7 days
	Patients with current HCC
**Anatomical criteria***	Portal vein thrombosis
	Anatomic abnormalities of the hepatic vasculature that prevent access to the intrahepatic portion of the portal vein or hepatic veins
	Evidence of active gastrointestinal bleeding
	If the volume of ascites in the path of the needle prevents apposition of the gastrointestinal tract and liver

* Identified at screening and/or during the endoscopic procedure
Abbreviations: INR, international normalized ratio; eGFR, estimated glomerular filtration rate; SBP, spontaneous bacterial peritonitis; HCC, hepatocellular carcinoma

Table S2. Primary and secondary study objectives

**Primary study objective**
Correlation between EUS-PPG obtained using the EchoTip® Insight™ and the HVPG performed simultaneously under general anesthesia
**Secondary study objective**
a. Comparison of the EUS-guided PVP (using the EchoTip® Insight™) to the transjugular WHVP
b. Comparison of the EUS-guided HVP (using the EchoTip® Insight™) to the transjugular FHVP
c. Evaluation of technical success (ability to take three measurements of the HVP and PVP using the EchoTip® Insight™ system)
d. Procedure-related adverse events
e. Evaluation of the association between EUS-guided measurements with clinical and lab-based features of portal hypertension such as varices, portal hypertensive gastropathy, thrombocytopenia, and liver elastography (if available within 1 year prior to procedure).
f. For patients undergoing TIPS placement: EUS-guided PVP (using the EchoTip® Insight™) is compared to the transjugular direct PVP obtained during the procedure
Abbreviations: EUS, endoscopic ultrasound; PPG, portal pressure gradient; HVPG, hepatic venous pressure gradient; PVP, portal venous pressure; WHVP, wedged hepatic venous pressure; HVP, hepatic venous pressure; FHVP, free hepatic venous pressure; TIPS, transjugular intrahepatic portosystemic shunt

Table S3. Differences in pressures depending on location of EUS-guided pressures.

	EUS-PVP measured in the		
	**Left portal vein branch (n = 13)**	**Main portal vein (n = 8)**	p-value*
EUS-PVP	24.6 ± 6.5	22.4 ± 5.3	0.42
EUS-PPG	11.2 ± 6.6	10.3 ± 3.5	0.71
	**EUS-HVP measured in the**		
	**Left hepatic vein (n = 11)**	**Middle hepatic vein (n = 10)**	p-value*
EUS-HVP	13.2 ± 3.8	12.6 ± 4.4	0.73
EUS-PPG	11.6 ± 6.3	10.0 ± 4.7	0.51

*Comparison of variances was performed using the F-test.
Numbers represent means ± standard deviations.
Abbreviations: EUS, endoscopic ultrasound; PPG, portal pressure gradient; PVP, portal venous pressure; HVP, hepatic venous pressure

Table S4: Disparity in hepatic venous pressure measurements (post hoc analysis)

	**|HVP − FHVP| < 3 mmHg** n = 10	**|HVP − FHVP| ≥ 3 mmHg** n = 11	**p-value***
**Baseline variables**			
Child-Pugh score	6.6 ± 1.7	8.2 ± 1.9	0.06
MELD score	9.5 ± 2.0	11.9 ± 5.4	0.43
BMI	27.4 ± 7.4	24.7 ± 4.7	0.34
**Hepatic vein GI access point (EUS)**			
Left hepatic vein	70.0%	36.4%	0.12
Middle hepatic vein	30.0%	63.6%	
**Hepatic vein assessment (EUS)**			
Depth of liver parenchyma traversed (mm)	36.6 ± 18.5	30.9 ± 8.8	0.38
Distance between hepatic vein puncture and IVC ostium	22.4 ± 8.1	14.2 ± 10.5	0.09
Diameter of the target segment of the hepatic vein (mm)	4.8 ± 1.8	7.1 ± 9.1	0.44

* Unadjusted raw p-value. All p-values in the current table are not statically significant after correction using the Hochberg procedure.
Numbers represent means ± standard deviations.
Abbreviations: HVP, hepatic venous pressure; FHVP, free hepatic venous pressure; MELD, model of end stage liver disease; BMI, body mass index; TIPS, transjugular portosystemic shunt; GI, gastrointestinal; EUS, endoscopic ultrasound; IVC, inferior vena cava

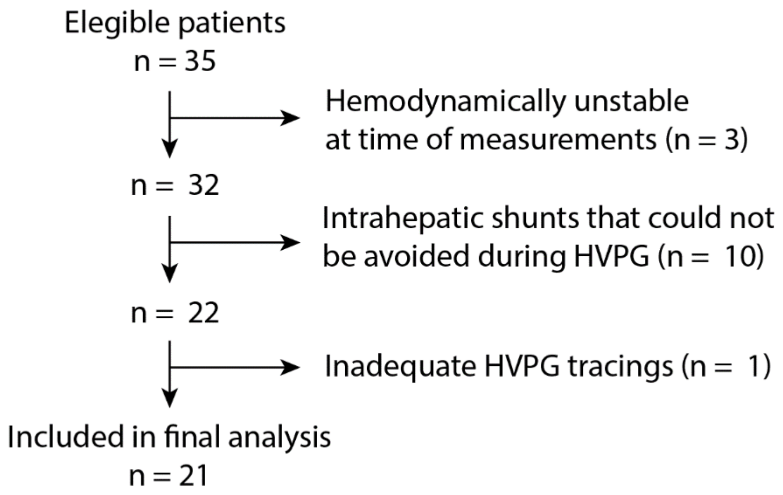

Fig. S1. Inclusion and exclusion flowchart

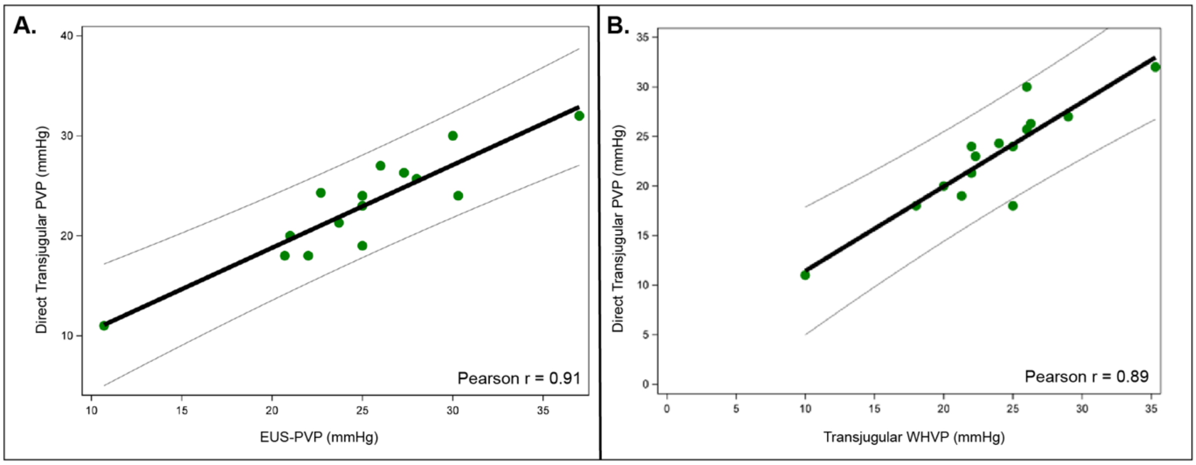

Fig. S2. (A) Correlation of direct transjugular portal pressure vs. EUS-PVP in patients receiving TIPS, Pearson r = 0.91, p <0.0001; (B) Correlation of the direct transjugular portal pressure vs. transjugular WHVP in patients receiving TIPS, Pearson r = 0.89, p <0.0001.

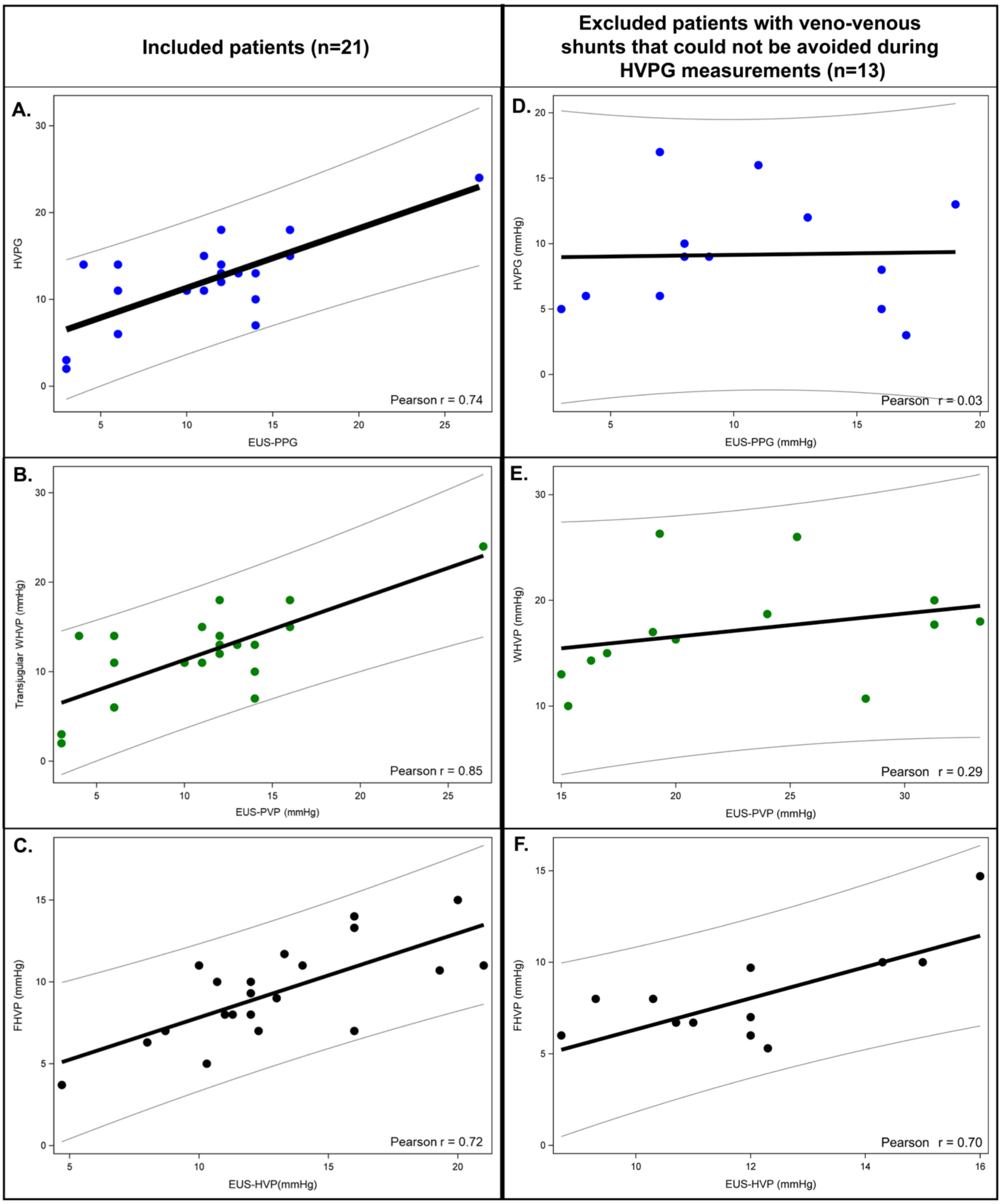

Fig. S3. Comparison of Pearson correlations for included patients (A, B and C) and patients excluded because of intrahepatic veno-venous shunts that could not be avoided during HVPG measurements (D, E, F) (all pressure were measured under general anesthesia). (A/D) HVPG vs EUS-PPG, (B/E) transjugular WHVP vs. EUS-PVP, (C/F) transjugular FHVP vs EUS-HVP.
